# Ensemble clustering of longitudinal bivariate HIV biomarker profiles to group patients by patterns of disease progression

**DOI:** 10.1007/s41060-022-00323-2

**Published:** 2022-05-04

**Authors:** Miranda L. Lynch, Victor DeGruttola

**Affiliations:** 1 Hauptman-Woodward Medical Research Institute, 700 Ellicott Street, Buffalo, NY 14203 USA; 2 Department of Biostatistics, Harvard T. H. Chan School of Public Health, 677 Huntington Avenue, Boston, MA 02115 USA

**Keywords:** Ensemble clustering, Shape-respecting distances, Dynamic time warping, HIV biomarkers, HIV disease progression

## Abstract

This paper describes an ensemble cluster analysis of bivariate profiles of HIV biomarkers, viral load and CD4 cell counts, which jointly measure disease progression. Data are from a prevalent cohort of HIV positive participants in a clinical trial of vitamin supplementation in Botswana. These individuals were HIV positive upon enrollment, but with unknown times of infection. To categorize groups of participants based on their patterns of progression of HIV infection using both biomarkers, we combine univariate shape-based cluster results for multiple biomarkers through the use of ensemble clustering methods. We first describe univariate clustering for each of the individual biomarker profiles, and make use of shape-respecting distances for clustering the longitudinal profile data. In our data, profiles are subject to either missing or irregular measurements as well as unobserved initiation times of the process of interest. Shape-respecting distances that can handle such data issues, preserve time-ordering, and identify similar profile shapes are useful in identifying patterns of disease progression from longitudinal biomarker data. However, their performance with regard to clustering differs by severity of the data issues mentioned above. We provide an empirical investigation of shape-respecting distances (Fréchet and dynamic time warping (DTW)) on benchmark shape data, and use DTW in cluster analysis of biomarker profile observations. These reveal a primary group of ‘typical progressors,’ as well as a smaller group that shows relatively rapid progression. We then refine the analysis using ensemble clustering for both markers to obtain a single classification. The information from joint evaluation of the two biomarkers combined with ensemble clustering reveals subgroups of patients not identifiable through univariate analyses; noteworthy subgroups are those that appear to represent recently and chronically infected subsets.

**Supplementary Information:**

The online version contains supplementary material available at 10.1007/s41060-022-00323-2.

## Introduction

Many diseases have characteristic molecular and cellular processes that define patients’ movement through their disease course; biomarkers that characterize these processes are often used to establish prognosis and to guide treatment. Individual trajectories of biomarker profiles over time are informative about disease progression, but combining information from multiple marker profiles from a patient population is challenging. The goal of this paper is to use cluster methods to evaluate patient groups in terms of their joint biomarker profile behavior. Cluster analysis, a widely used unsupervised machine learning tool, investigates whether a collection of objects can be grouped into cohesive subsets, as grouping structure can shed light on the processes that underpin group membership. We focus on cluster methods for trajectory or curve data. Standard methods for multivariate data clustering used for non-trajectory data are not designed to accommodate within-profile dependence structures, sequential ordering of time points, or global and local curve behavior.

We use cluster methods to detect distinct progression types based on similar biomarker trajectory patterns. To achieve this goal requires methods that can group individuals based on the overall shape of their profiles; such grouping requires shape-respecting distances. Shape-respecting distances are important in clustering disease progression trajectories, as they capture overall patterns of behavior while providing the flexibility needed to handle irregular times of measurement, variable numbers of profile observations, and retain time order of observations. We then use ensemble cluster methods to combine the information derived from univariate clustering to reveal joint behavior of multiple profile types. The goal is to combine across multiple trajectory profiles to evaluate patterns of disease progression that are not detectable from univariate clustering. We first use the shape-respecting distances in generating individual input dendrograms for each biomarker under study, and then aggregate the input dendrograms of each biomarker to a single output clustering that synthesizes the information of the individual biomarker-level clustering. Our application makes use of data from a cohort of Human Immunodeficiency Virus (HIV)-infected patients with unknown time of infection, who were recruited at different disease stages and times from initial infection. Our goal is to identify patients whose disease progression follows a similar course (although perhaps at different speeds) via our ensemble clustering approach.

The HIV biomarkers we model, CD4 T-lymphocyte count (CD4) and viral load (VL), are commonly used for HIV monitoring, and are often used in tandem to ascertain disease progression, estimate time of treatment initiation, and evaluate treatment efficacy [1, 2]. CD4 T-cells belong to a class targeted by HIV and provide information on disease course. Our methods address heterogeneity in disease progression that allows individuals to be grouped into subsets based on marker trajectories [3]. Categories of HIV progression that have been reported include fast-progressors, durable non-progressors and typical progressors. There has been considerable research effort to distinguish between groups of fast and slow progression to better inform treatment decisions [4, 5]. Our goal is to characterize heterogeneity in HIV disease progression and to identify distinct patient subgroups. Such investigations are complicated by lack of available models that can accommodate highly variable trajectories and lack of knowledge regarding infection times. Profile clustering techniques for identifying trajectory clusters mostly depend on regularly spaced data, as are typically observed in time series [6, 7]. Such data are not generally available for patients in HIV studies, due to participant drop-out, missed measurements and irregularly spaced measurement times, but the degree of departure from the regularly spaced ideal varies across studies. Here, we investigate methods that allow us to cluster biomarker trajectory data into subgroups using two related shape-respecting distances, Fréchet and dynamic time warping (DTW), each of which accommodates missing measurements and irregular spacing, and importantly, preserves the time ordering of the data. Use of a distance that incorporates profile shapes (which depend on observation ordering), while allowing for flexibility in number and spacing of observations, enables assessment of similarity of profiles without the need for exact time referencing.

Dealing with sparseness in marker measurement data and its impact on investigation of viral load effects on transmission risk is also an important challenge in HIV, SARS CoV-2 and other infections. There can be major departures from typical progression for different reasons such as exposure to other pathogens and/or host genetic background, in addition to disease stage. Understanding factors that induce different progression patterns is a key question in HIV research, as such patterns impact choices of treatment strategies [4, 5]. Standard approaches for analyzing trajectory data, such as random effects models, permit a regression approach that can shed light on determinants of trajectory behavior, but generally require restrictive assumptions regarding smoothness and regularity of trajectories, including strong distributional assumptions on error structure. Thus, direct application of mixed effects models to trajectory profile data can be difficult when latent subgroups are present. We investigate a clustering approach based on between-trajectory distances to delineate trajectory subgroups in the set of profiles without the need for strong parametric assumptions.

## Related work: trajectory clustering

Many clustering methods rely on defining distances as a way of quantifying the level of similarity between the objects to be clustered (in our case, longitudinal and trajectory data). Partitional clustering such as k-means for trajectory data uses distances between profiles and iterative cluster centers, such as has recently been demonstrated by Genolini, who implemented an algorithm in R [8, 9]. In hierarchical clustering methods, all pairwise distances between objects are computed, and agglomerative or divisive methods act on the distance matrix. The most widely used methods for computing inter-profile distance are based on Minkowski-type distances for objects in a normed vector space—in particular Euclidean and Manhattan distances. These distances require that all profiles be of the same length to be computed, and are invariant to order permutations [10, 11]. Ordering of values should be a key component of the inter-profile distances, as ordering gives each profile its characteristic ‘shape.’ We consider elastic distance measures Fréchet [12] and dynamic time warping (DTW) [13] for the reasons described above.

We focus on the related Fréchet and DTW distances for profile clustering, as recent comparisons of performance of elastic distance measures such as DTW in time series show very favorable performance over alternatives [14]. These distances have been used for comparisons of time series, longitudinal data, and in functional data analysis [13, 15–17], in applications such as curve comparisons in household energy consumption [18], GPS track data [19], and EEG signals in neuroscience [20].

Fréchet distance and DTW both rely on acceleration/deceleration transforms of the time axis to identify similar trajectory shapes with similar patterns over time that differ in relative timing. We first describe Fréchet distance to establish notation and describe ordering, and then present DTW as a related distance. For continuous curves *f* and *g* in a metric space equipped with a distance *d* (possibly each defined over different intervals), the continuous Fréchet distance between *f* and *g* is given by:

1 $$\begin{aligned} \delta _{contF}(f, g) = \inf _{\alpha , \beta } \max _{t \in [0,1]} d(f(\alpha (t)), g(\beta (t))), \end{aligned}$$

for $\alpha , \beta$ arbitrary continuous nondecreasing functions from [0, 1] to the respective intervals of *f*, *g*.

Discrete Fréchet distance is defined for polygonal curves, where each curve is represented with a sequence of connected line segments, and only uses distances between the nodes of the curves in its construction. It relies on defining a *coupling* *L* between the curves *P* and *Q* with nodes at $(u_1, \ldots , u_p)$ and $(v_1, \ldots , v_q)$, as the sequence, $(u_{a_{1}}, v_{b_{1}}), (u_{a_{2}}, v_{b_{2}}), \ldots , (u_{a_{m}}, v_{b_{m}})$ of distinct pairs from each node set subject to the following index constraints:

$$\begin{aligned}&a_{1} = 1, a_{m} = p \ \ b_{1} = 1, b_{m} = q\\&a_{i+1} = a_{i} \textit{ or } a_{i} + 1\ \ b_{i+1} = b_{i} \textit{ or } b_{i} + 1 \end{aligned}$$

These index constraints preserve ordering of the measurements along the curve. The notion of a coupling has a parallel in the definition of DTW as well. Defining the length $\Vert L\Vert$ associated with the coupling *L* as the longest distance between points in the coupling gives the definition of the discrete Fréchet distance as:

2 $$\begin{aligned}&\delta _{discF}(P, Q) = min\lbrace \left\| L\right\| \text { for } L \nonumber \\&\quad \textit{ a coupling of P and Q} \rbrace . \end{aligned}$$

for all possible couplings subject to the above constraints. This distance defines a discrete metric on the set of polygonal curves that provides an upper bound to the continuous Fréchet distance, and is efficiently computable in *O(pq)* runtime via a dynamic programming algorithm [21–23]. Fréchet distance is considered a shape-based distance, based on its reliance on a maximum which emphasizes geometric features of the trajectories being compared [24].

Related to discrete Fréchet distance is dynamic time warping (DTW), which determines an optimal mapping between two time series (not necessarily of equal number of time points) by ‘warping’ the two series vectors onto a set of points such that the summed distance between them is minimized [13, 25, 26]. This allows for local elastic stretching and compression of the time sequences so that similar shapes that occur with difference in timing or phase can be detected and aligned. The DTW algorithm defines a warping path between two polygonal curves *P* and *Q* that aligns the elements of each, subject to boundary and continuity constraints that are similar to those described above for Fréchet. The DTW method then selects the warping path that minimizes the *cumulative* distance (typically using Euclidean distance as the local similarity metric) over the path between *P* and *Q*. Thus,

$$\begin{aligned} \delta _{DTW}(P, Q) = \min _{W} \left[ \sum _m d(w_m) \right] , \end{aligned}$$

where *W* is a warping path $$ (w_1, \ldots , w_m) $$, each $$ w_m $$ is an $$ (i,j)_k$$ element in the alignment of the elements of *P *with the elements of *Q*, $$ d(w_m) $$ is a distance between the curves at vertices *i* and *j*, and the minimum is taken over all possible paths *W*. The warping function *W* for DTW aligns the time indices of *P* and *Q* such that time deformations result in the curves being brought as close together as possible, under a monotonicity constraint that retains the ordering of the points. Thus, the DTW distance is very similar to discrete Fréchet, with warping curve analogous to the coupling described above, but the former locates a minimum sum between aligned curve vertices, rather than the minimum maximum distance (‘least of the longest,’ across all couplings) between a single pair of vertices.

### Simulation study to compare performance of shape-respecting distances

To evaluate the performance of shape-respecting distances in clustering trajectory data of varying shapes subject to different levels of missingness, we carried out an empirical investigation of Fréchet and DTW distances for clustering a benchmark dataset of simulated time series profiles. We note a recent related analysis of trajectory similarity measures that does not consider missing data [11]. We used the cylinder–bell–funnel (CBF) benchmark data—a set of simulated profiles of different shapes widely used to examine performance of classification and clustering algorithms [27–29]. The data consist of vectors of 128 equispaced simulated noisy measurements from three profile shapes, pre-split into a training set (*n*=30) and a test set (*n*=900); example profiles illustrating the shapes are given in Supplemental File 1. In supervised learning classification applications of the CBF data, the training set is used to train a classifier. In the context of clustering, there is no supervised learning or requirement for separate test/train sets. We used only the training set of profiles in our simulation to examine the degree of shape clustering, and to assess the impact of simulated missingness on performance under the Fréchet and DTW distances. Multiple implementations of both similarity measures are available, each of which can have different performance and run times. We selected implementations that were most equivalent in terms of weighting and handling of the time axis, to facilitate comparison. We used the Fréchet metric as implemented in the R package *kml* (v 2.4.1) [8], and the DTW distance as implemented in R package *dtw* (v 1.20-1) [25].

We simulated missingness in the CBF benchmark data under an assumption that data are missing completely at random (MCAR) for 2 preselected levels of missingness (removal of 25 and 50 percent of data). We used two forms of the CBF training set, the ‘full’ data, wherein each profile had the full 128 data points prior to imposed missingness, and a ‘sparse’ version of the CBF training set, wherein each fourth data point was used. Each sparse profile had 32 equispaced points prior to imposing missingness. We chose a sparse set to reflect the wide variability in the numbers of longitudinal observations seen in practical applications, including our own application area. We generated profile missingness by removing the appropriate number of data matrix positions at random and without replacement from the matrix of measurement values. This missingness pattern results in some profiles with few or no missing observations and others with considerably more. A plot of one instance of the simulated missing data for the sparse CBF profile set is provided in the supplementary material [see Supplemental File 2].

**Table 1:** CBF benchmark data simulation results. Summary information on cluster evaluation metrics for different levels of imposed missingness in the full and sparse versions of the CBF benchmark profile dataset. Each nonzero level of missingness was evaluated in *n*=500 simulated datasets, with results given as mean (sd); Results on the single no missingness dataset for the full and sparse CBF provided for reference

Data, % missing	Distance	AdjRand	Fowlkes-Mallows	Purity
CBF Full data,	Euclidean	0.139	0.494	0.633
0% (for reference)	Fréchet	–0.028	0.438	0.400
	DTW	0.486	0.705	0.633
CBF Full data,	Fréchet	0.268 (0.164);	0.548 ( 0.092)	0.621 (0.106)
25%	DTW	0.397 (0.164)	0.630 (0.109)	0.670 (0.093)
CBF Sparse data,	Euclidean	0.174	0.446	0.600
0% (for reference)	Fréchet	0.110	0.419	0.567
	DTW	0.463	0.682	0.733
CBF Sparse data,	Fréchet	0.215 (0.162)	0.531 (0.081 )	0.606 (0.103)
25%	DTW	0.529 (0.246)	0.709 (0.143)	0.773 (0.141)
CBF Sparse data,	Fréchet	0.106 (0.127)	0.493 (0.061)	0.529 (0.086)
50%	DTW	0.298 (0.186)	0.572 ( 0.103)	0.651 (0.112)

**Fig. 1:**
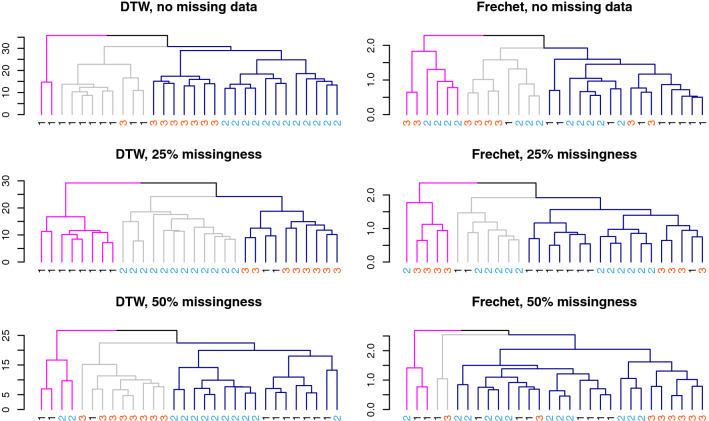
Representative clustering of benchmark CBF data under missingness. Clustering of single sparse set of CBF benchmark data under different distance and missingness levels, to illustrate performance of DTW and Fréchet distances in capturing the known shape labels. Branch colors highlight the cluster results, leaf labels and colors show true group identity. The single sparse set with no imposed missingness is represented in the top level figures. A single simulated sparse dataset with 25% and 50% missing observations are represented in the second and third levels of figures, respectively

#### Performance evaluation on benchmark CBF data

Performance evaluation of the two distances in clustering labeled benchmark data relied on external cluster validation criteria that provide information on how well the known CBF shape group labels are captured. We selected Adjusted Rand Index, the Fowlkes–Mallows Index, and purity measure to evaluate the agreement between the partitions generated under each distance measure and the benchmark CBF groupings. For these evaluations, the partitions generated under the two distance metrics for a given simulated dataset (i.e., a set of CBF profiles with a given level of randomly generated missingness) is compared to the partition arising from the known labels. The objects being clustered are the *n*=30 profiles, labeled with their profile shape. The Rand Index [30] uses the fraction of counts of correctly classified pairs of elements relative to total number of possible pairs in the clusters, and serves a basis for the adjusted Rand Index [31], a corrected-for-randomness extension that accounts for random appearances of pairs under a generalized hypergeometric distribution model for the randomness. Fowlkes–Mallows Index [32] is the geometric mean of precision and recall from information retrieval, with higher values indicating greater recovery of the benchmark labeling by the clustering procedure. The purity measure for clustering relies on a measure of intra-cluster group similarity for a given partition. It gives a measure of the extent to which each of the cluster groups contains objects of a single class [33]. Values near 0 imply poor clustering performance, while a purity measure of 1 indicates a perfect performance. We compute these indices for each of the simulated datasets for both the Fréchet and DTW distance-based clusterings, and report summary information across the simulated datasets in Table 1. An example of results from one cluster simulation for each level of imposed missingness for the CBF data appears in Figure 1. These results imply that relative to DTW, there tends to be lower variability in the Fréchet indices–and hence reduced diminishment of performance—as more data points are removed. Nevertheless, the overall performance for capturing known cluster labels strongly favors DTW at all levels of imposed missingness. We note the poor performance of standard Euclidean distance relative to DTW for comparison in the no-missingness case where it is able to be computed, due to its inability to accommodate misaligned but similarly shaped profiles. (Although interestingly, it outperforms Fréchet in the full dataset, likely due to the use of more of the information in the data, even though misaligned.) While DTW outperforms Fréchet in our benchmark dataset, relevant to our application of varied-length biomarker trajectories, theoretical guarantees of performance that would favor a given metric are generally lacking, thus requiring relevant empirical comparisons for selecting between metrics or methods [34].

There is an extensive literature on analyzing and comparing distance measures applied to trajectories, time series, speech patterns and other data in the form of profiles (see [11, 35–37]). The primary difference between these two distance measures is the number of quantities used to construct the profile-wise difference measure. The acceleration/deceleration transformations inherent in the Fréchet distance, as well as the warping path used in DTW computation, would be expected to make these shape-respecting metrics robust to effects of missing data. We observe that DTW outperforms Fréchet in terms of recovering known shape labels, but the effect diminishes as missingness increases. This happens because Fréchet relies on a single distance between a specific pair of vertices; random missingness that does not directly impact that distance does not change the value. DTW relies on multiple distances via a sum, so the summed distance is altered as missingness increases. For all three performance measures, the value gap favoring DTW over Fréchet shrinks with increasing amounts of missing data. The alignment feature (the warping path for DTW, or the coupling that drives the acceleration/deceleration of the Fréchet distance) is key to preservation of observation ordering. Fréchet uses only a small portion of the information from the alignment—the single maximum distance for each possible coupling of curve vertices—and then minimizes that set of maxima. The DTW warping curve uses an optimization that employs information from multiple vertices, and thereby makes use of much more shape information, resulting in better cluster performance even as missingness increases.

## Ensemble clustering for multiview longitudinal data

We next address how to use ensemble clustering methods to combine the information from multiple biomarkers. Most work in consensus or ensemble clustering (both terms are used in the literature) has focused on ensembling to improve the performance of individual clusterings. A primary motivation for consensus methods is to enhance the quality or robustness of the consensus result over that of the individual base clusterings, with most base clusterings arising from multiple runs of the clustering algorithm (for instance, several runs of k-means with different starting centroid positions, or different values of *k*). Also, most of it has focused on partitional clustering such as arise in k-mean-type clustering methods (see [38, 39]), with much less attention paid to ensembling of hierarchical cluster results. Recent work addressing consensus of hierarchies includes [40, 41] and [42]. Use of ensemble methods for *multiview* clustering, where a single dataset provides multiple measurement sets (‘views’) from different measurement modalities, appears less frequently as an application. Ensemble methods in the multiview case proceed with the different goal of finding a single unified clustering that best synthesizes the information content in each of the multiple views. In this framework, each view is a base clustering, and the goal of ensemble methods is to summarize the multiple views into a single clustering. We focus on agglomerative hierarchical clustering to generate the base clusters, and the ensembling combines the information contained in the base dendrograms.

Unlike ensemble clustering for partitions, which acts on the sets of cluster labels in each base clustering, ensembling of hierarchical clusters can use the information contained in the full dendrograms. Hence, it is not necessary to ‘cut’ the dendrogram and generate class labels, but only to specify the set of input dendrograms. Since a hierarchy is a rooted, node-indexed nested tree, the information in the dendrogram includes the nesting pattern and the heights at which different branches merge during clustering; the ensembling process we discuss operates on this information content to define distances between the dendrograms themselves. In combining multiple hierarchies, we desire methods that use ultrametrics for the ensemble process, to ensure unique reconstruction of the terminal consensus result. We provide here a brief discussion of metric and ultrametric spaces to motivate our work using shape-respecting distances and ensemble methods. A *metric* space (*X*, $\delta$) is a set *X* of points coupled with a distance $\delta$, where the distance has the following properties:

1. *Non-negativity*: $$\forall x_i, x_j \textit{ in } X, \delta (x_i, x_j) \ge 0 $$
2. *Symmetry* : $$ \delta (x_i, x_j) = \delta (x_j, x_i) $$
3. *Separation*: $$ \delta (x_i, x_j) = 0 \iff x_i = x_j $$
4. *Triangle inequality*: $$\forall x_i, x_j, x_k \textit{ in } X$$,
  $$\begin{aligned} \delta (x_i, x_k) \le \delta (x_i, x_j) + \delta (x_j, x_k) \end{aligned}$$

In an *ultrametric* space, the distance requires the more restrictive *ultrametric inequality*, as a stronger version of the triangle inequality:

$$\begin{aligned} \delta (x_i, x_k) \le max(\delta (x_i, x_j), \delta (x_j, x_k)), \forall i, j, k. \end{aligned}$$

We note that we can weaken the third property above to the following, resulting in a pseudo-metric space: $3^{\prime }: x_i = x_j \Rightarrow \delta (x_i, x_j) = 0$.

The use of ultrametric distance for the consensus mechanism operating on individual dendrograms is key to providing a unique reconstruction of the ensemble dendrogram. For *each* hierarchical base clustering, merge heights and internal node structure reflect the strength of shape similarity between pairwise comparators (leaf nodes, which are biomarker profiles in our application).

To combine the individual dendrograms requires characterizing their dissimilarities, and these dissimilarities (termed dendrogram descriptors or distances) are distances derived from the dendrogram features themselves. Each hierarchical clustering of *N* inputs can be associated with an $N \times N$ matrix that portrays the relative leaf node positions in the dendrogram. Examples of dendrogram descriptors include cophenetic distance in which the lowest merge distance for two leaves is used as node dissimilarity, or cluster membership divergence, in which dissimilarity is taken as the smallest cluster size in the dendrogram that contains two specified leaf nodes [40]. These dissimilarities form the basis of aggregation. We state the following:

*Proposition*: There exists a bijection between an agglomerative hierarchy formed on set *X* using a given agglomerative procedure *C*, and an ultrametric space, that is, given a totally indexed hierarchy *H* on the set *X*, we can define an ultrametric distance $\delta$ satisfying the properties above (including the ultrametric inequality); furthermore, there exists an ultrametric space (*X*, $\delta$) with distance $\delta$ such that *H* can be exactly recovered. See [43, 44] and references therein for more thorough discussion and proofs.

The above proposition ensures that we can uniquely generate an ensemble clustering from agglomerative input dendrograms by employing aggregation methods that result in an ultrametric distance for generating the consensus. Aggregation of dendrogram descriptor matrices occurs by locating an ultrametric that is ‘closest’ to the *m* input ultrametric descriptor matrices, via minimizing a squared distance (typically Euclidean) to the collection of the *m* dissimilarities inherent in the input dendrograms. This step usually proceeds via heuristics, although explicit solutions to the least squares problem are available in some special cases (see [45] for details). We chose the SUMT (Sequential Unconstrained Minimization Technique) approach of de Soete [46] for carrying out this minimization, as implemented in the *CLUE* (*CLU*ster *E*nsembles) package [45, 47] in the R statistical software environment [48]. Thus, the input hierarchies are rendered ultrametric via converting them to descriptor matrices (in our case, via cophenetic distance), and a consensus ultrametric is determined via locating the least squares minimization of the descriptor matrices to a final ultrametric. These dendrogram descriptors form a sort of intermediate toward the final ultrametric construction, which itself is derived via an optimization. Finally, based on the proposition, this final ultrametric can then be used to uniquely recover the consensus dendrogram result.

DTW is not a metric distance, since it fails the triangle inequality [49]. (Although it is worth noting that in actual practice, failures of the triangle inequality are extremely rare, see [50].) Hence, a space of points (in our case, trajectories) accompanied by DTW as a distance does not form a metric (or thus ultrametric) space. Rather, DTW acts as a measure of dissimilarity between two curves (satisfying other properties of a metric, but lacking triangle inequality). Nonetheless, we can still form a hierarchical clustering using DTW as (nonmetric) distance. Below, we derive a set of dendrograms, one each for our univariate biomarker profile sets. These hierarchies can be mapped (based on the proposition above) to an ultrametric space. In the multiview setting of this work, the hierarchical agglomerative algorithm operates on the same set of observations (patients in our example), but uses multiple (non-metric) dissimilarity matrices (one for each biomarker view). The main idea in our approach is to construct the ultrametric used for the consensus result from the dendrograms for the biomarker data. By constructing the ensemble from the dendrograms—not from the original matrices of DTW distances for each biomarker—we can derive a unique ensemble. Thus, we can form an ultrametric consensus matrix *D* constructed from hierarchies derived from nonmetric distances that are aggregated using dendrogram descriptors [40]. The final outcome is a consensus that reflects the hierarchies of the original base clusterings for each biomarker. These input clusterings use shape-based distances for the trajectories.

## Application and results

We apply the methods for ensemble shape-based clustering to a set of longitudinal HIV biomarker profiles. Profile HIV biomarker data for our study of HIV progression is derived from a randomized, double-blind placebo-controlled clinical trial conducted in Botswana to investigate whether micronutrient therapy delayed disease progression in a prevalent cohort of treatment-naïve HIV positive individuals [51]. In that study, supplementation was found to have no effect on viral load, although was well-tolerated in the study population. Study participants were enrolled between December 2004 and July 2009, with eligibility criterion of CD4 cell count >350 cells/$\mu$L at enrollment. Study nominal time period was 24 months, with scheduled biomarker assessment every 6 months (scheduled measurements at months 0 (baseline assessment), 6, 12, 18, and 24). Patient actual study times ranged from 0 to 25 months recorded in whole month increments for this analysis, and deviations from the set visit schedules were common. We restricted attention to individuals who had 4 or more visits over the time period, resulting in *n*=646 individuals available for the clustering analyses from the original cohort (*n*=875). The mean (median) number of visits per patient in the original cohort was 4.2 (5), with a range from 1 to 7; for the analyzed subset, mean (median) number of visits was 4.8 (5).

We use shape-respecting distances to examine HIV biomarker profiles for which times of infection that would ‘anchor’ observations in time are unknown. Our analyses examine the *relative* behavior of biomarker profiles in a way that preserves measurement ordering and emphasizes profile shapes. This choice of analysis is motivated by the notion that individuals will experience similar patterns of disease progression, possibly at differing levels of intensity and timing, which are best captured by grouping participants with similar trajectory shapes even if they differ in timing of disease course.

### Univariate profile clustering for HIV biomarker data

**Fig. 2:**
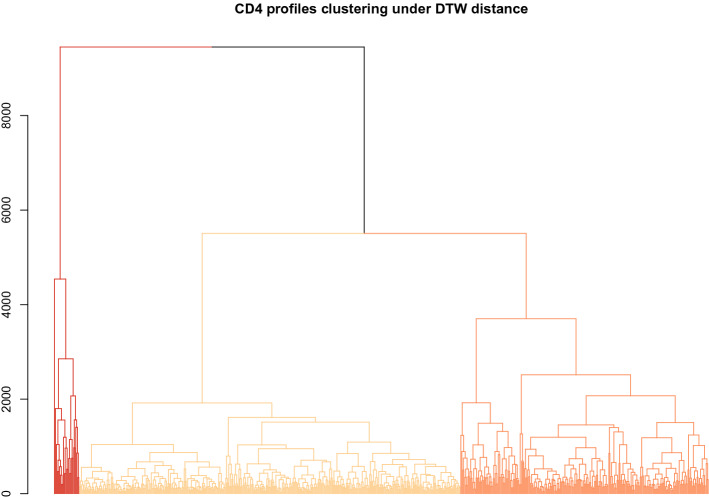
Univariate clustering results for the Dikotlana CD4 profile measurements under the DTW distance measure, showing a three-group clustering. Cluster groups are shown with the lowest CD4 profile group in the lightest shade, and the highest CD4 profile group in the darkest shade. Plots of patient CD4 profiles in each of the clusters are shown in Fig. 4

**Fig. 3:**
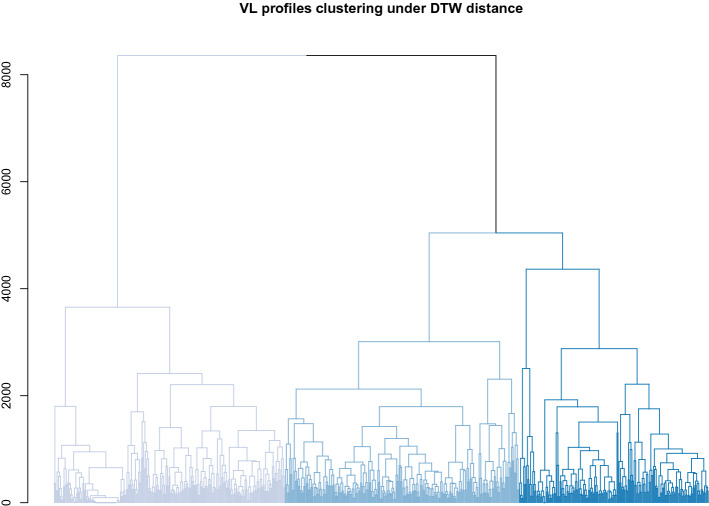
Univariate clustering results for the Dikotlana log$_{10}$ VL profile measurements under the DTW distance measure, showing a three-group clustering. Cluster groups are shown with the lowest log$_{10}$ VL profile group in the lightest shade, and the highest log$_{10}$ VL profile group in the darkest shade. Plots of patient log$_{10}$ VL profiles in each of the clusters are shown in Fig. 5

**Fig. 4:**
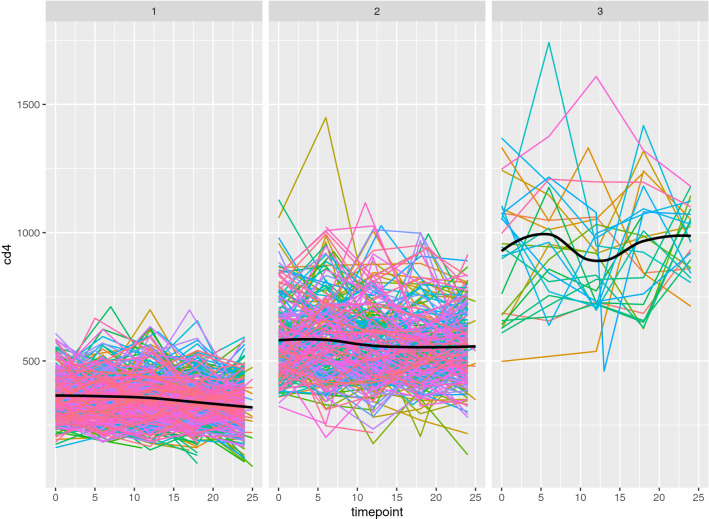
Patient CD4 profiles by cluster group, corresponding to the clusters shown in Fig. 2. Each individual longitudinal profile is shown in a single color. When possible each individual profile is shown using a unique color; larger clusters require reuse of the same color for multiple individuals. A loess smooth (thick black line, computed on original time scale for each panel) is added to highlight the overall trend within each cluster. Times are in months

Pairwise DTW distances between each pair of profiles within each biomarker set were computed in the R statistical software environment [48], using the DTW package (v 1.20-1, [25]). The resulting distance matrices were clustered using complete linkage hierarchical clustering using the *hclust* function available in base R. The standard log$_{10}$ transformation was used prior to distance calculations and cluster analysis for the VL values. Results for the CD4 and VL biomarkers appear in Figs. 2 and 3, respectively. From these analyses, three primary CD4 clusters are observed. The largest cluster includes profiles from 376 of the 646 participants; these profiles are observed to have slow, steady declines with relatively little variability and CD4 cell counts consistently below 500 (see Fig. 4).

**Fig. 5:**
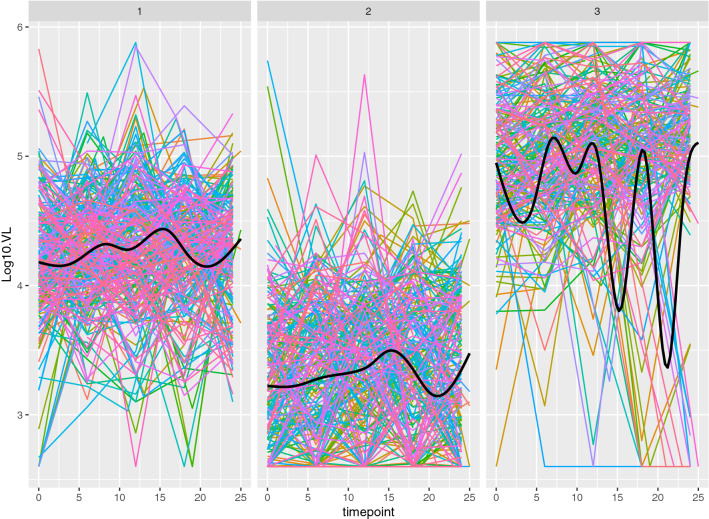
Patient log$_{10}$ VL profiles by cluster group, corresponding to the clusters shown in Fig. 3. Each individual longitudinal profile is shown in a single color. When possible each individual profile is shown using a unique color; larger clusters require reuse of the same color for multiple individuals. A loess smooth (thick black line, computed on original time scale for each panel) is added to highlight the overall trend within each cluster. Times are in months

Results for the VL biomarker are less straightforward. Figure 5 shows VL profiles over time broken out by cluster groups, and highlights several important results. The VL measurements are highly variable and subject to both upper and lower quantitation limits (400 and 750,000 counts, respectively). There were 228, 231, and 187 patients, for the low, medium, and high VL profile group clusters, respectively. The first two of these groups showed clear increasing levels of viremia, although both had a pronounced dip in viremia near the end of the study period (Fig. 5).

Group overlap between CD4 and VL cluster groups is shown in Table 2. A basic $$ \chi ^{2} $$ test examining the counts of patients falling into different patterns of CD4 and VL profile shapes shows a highly significant association between the profile types ($$\chi ^{2}$$ = 53.4 on 4 df, p $$<<< $$ 0.001). Cluster validity was examined using multiple criteria (Dunn index, Silhouette index) that provide information on cluster stability in unlabeled data [52]. Results are given in Table 3, generated using the *fpc* package in R [53]. Silhouette index registers within-cluster cohesion relative to between-cluster separation as a measure of cluster validity. As expected, this metric shows higher value for the CD4 than the VL clustering; the higher value reflects the lower noise level in CD4 counts. By contrast, the Dunn index values show similar magnitudes for both CD4 and VL profiles. This reflects the reliance of the Dunn index on a maximum (as opposed to average) within-cluster compactness measure, and implies that CD4 and VL are likely to have had similar maximum values for this measure.

**Fig. 6:**
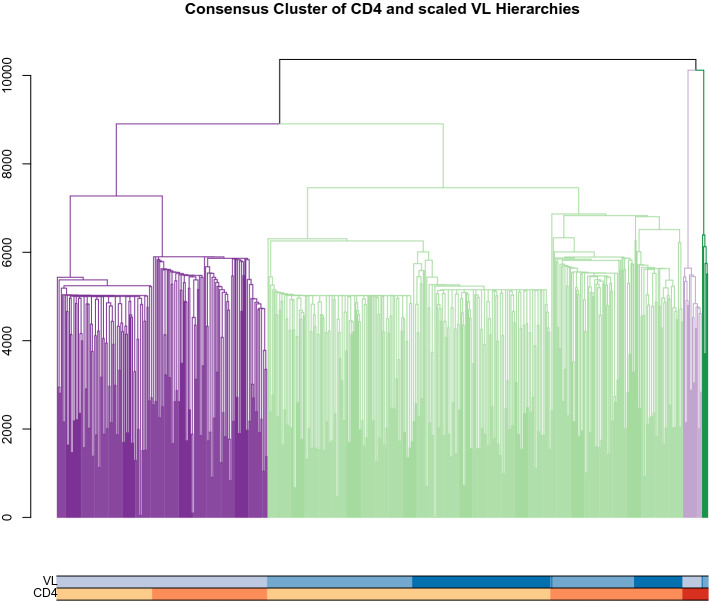
Dendrogram of ensemble cluster results for the HIV patient data, from combining the univariate cluster results of the log$_{10}$ VL and CD4 trajectories, showing a four-group clustering. Colored bars below the dendrogram show how the univariate cluster groups appear relative to the final consensus result. Univariate clusters are represented in the same color schemes as Figs. 2 and 3

Inspection of univariate clustering for both biomarkers reveals patterns that might be expected from the biology of HIV progression; we examined these patterns using loess smooths of the profile collections. It is important to note that the loess smooths on the grouped profiles were done after the cluster procedures, and carry out smoothing on the original timescale, whereas the DTW-based clustering employed warping of timescales for each profile in the distance calculations. We include the loess *ex post facto* for the purpose of highlighting trends and variability levels on the raw data scale. Among the CD4 cluster groups, the low CD4 cluster indicates the slow but steady decline in CD4 levels over the 2 year study period, whereas for the medium CD4 cluster group, profiles are more variable, are consistently at a higher CD4 level, and show a somewhat reduced decline over the study period. The third CD4 group, consisting of only 25 of the 646 profiles, had comparatively high CD4 levels, and showed strong within-individual profile variability. The high CD4 counts and irregular profiles imply that this was a small set of recently infected individuals who had not yet undergone pronounced CD4 decline. These patients in the high CD4 cluster are largely a subset of the individuals in the lowest VL group; notably this group is also a VL subcluster with VL profiles maintained near or below viral detection limits, as expected from the fact that CD4 cells are the virus targets. These considerations and the strong association between profile groups led us to investigate combining the two biomarker clustering results to arrive at a single partition of patients into progression subtypes based on information from both biomarker profiles.

### Ensemble clustering in HIV multi-marker data

We used ensemble clustering as a means of aggregating the hierarchical univariate clusterings presented in Sect. 4.1 to examine how the co-behavior of the markers jointly inform on disease progression. Frequently used approaches for examining bivariate marker measurements include modeling one marker as a function of the other, or joint modeling of the biomarkers. The latter requires assumptions about the trajectory models that are not likely to be met in our data. Our analytical goal is to examine how the bivariate trajectory patterns help identify groups at different stages of infection or that experience different patterns of progression.

**Table 2:** Cross-tabulation of counts of patients in log$_{10}$ VL cluster groups (rows) and CD4 cluster groups (columns). Groupings of low, medium, and high correspond to the plots of patient profiles in Figs. 4 and 5

	CD4 L	CD4 M	CD4 H
VL L	95	114	19
VL M	144	82	5
VL H	137	49	1

**Table 3:** Internal cluster validity measures for the univariate cluster results, for each biomarker

	Avg Silhouette	Dunn Index
CD4	0.449	0.024
VL	0.344	0.032

Ensemble or consensus cluster methods combine profile contributions at the level of the partition or hierarchy, thereby providing information on progression types at the level of the cluster groups. Joint clustering may not produce the same clusters as does individual level clustering. Our goal is to find the ‘best’ clustering that summarizes the information in multiple markers into a single consensus result. This is challenging, as the univariate base cluster results of the CD4 and VL biomarkers operate on different scales, and with different amounts of variability in their profiles. The base clustering inputs for ensembling were the DTW-based CD4 and VL cluster results. For the consensus methods, we rescaled the DTW distances obtained for the log$_{10}$ VL data, using a multiplicative scaling factor of 300. The rescaled distances do not impact the original base clustering, but render the VL results onto a similar scale and distribution of distances as CD4 profiles; this facilitates a nearly equivalent weighting of the two base clustering inputs. Consensus cluster results are shown in Fig. 6, colored with respect to a four-group clustering. We used the R implementation *CLUE* (v 0.3-57 [45, 47]) to carry out the computations. Information on the repositioning of patients into cluster groups, from comparing the univariate results for each biomarker with their grouping under the consensus result, are given in Tables 4 and 5. We note that there is a biological rationale for the final consensus results that supports the number (4) of clusters. In general, identifying the number of clusters remains a very active research question. Adragni et al. propose a method based on principle fitted components and sequential testing [54]; Kingrani et al. review multiple methods and propose an approach based on diversity measure [55].

**Table 4:** Cross-tabulation of univariate CD4 cluster groups with the ensemble clustering result

	Ens 1	Ens 2	Ens 3	Ens 4
CD4 L	281	95	0	0
CD4 M	131	114	0	0
CD4 H	0	0	19	6

**Table 5:** Cross-tabulation of univariate VL cluster groups with the ensemble clustering result

	Ens 1	Ens 2	Ens 3	Ens 4
VL L	0	209	19	0
VL M	226	0	0	5
VL H	186	0	0	1

An interesting result that emerges in the bivariate analysis using ensemble cluster methods is that the small group (*n*=25) of patients identified in the univariate CD4 analysis as having high CD4 readings are, in the bivariate analysis, more separated from the remaining patient profiles; evidence for this is provided by the large merge heights for that group relative to the other clusters. Thus, the ensemble results retain the high-CD4 cluster; but the incorporation of VL profile information yields a strong separation of that CD4 group into two distinct subgroups based on VL behavior—one with relatively low VL counts over time (*n*=19), and a small group (*n*=6) with higher VL profiles. This latter group has characteristics consistent with its being a recently infected subset. The prominent split of the ‘high CD4 profile cluster’ underscores the ability of the ensemble cluster method to identify prognostic groups using multiple sources of information.

The rearrangement of the larger groups also provides insight into relationships of interest. Figure 6 provides the results for both univariate base clusterings in colored bars beneath the consensus dendrogram. The ensemble results for the clusters of each marker treated individually show evidence of subgroupings that are defined by the clusters of the other biomarker. For example, in the ensemble cluster results for CD4 count shown in the dendrogram in Fig. 6, the leftmost cluster shown (shown in dark purple) consists of almost all of the participants who display lower and increasing VL values; furthermore, that VL behavior is the only pattern found in that ensemble cluster. That cluster itself shows fairly strong subclustering based solely on CD4; it is divided approximately evenly between participants with a low CD4 level and those with an intermediate CD4 level; the merge height provides strong evidence for this subclustering. The phenomenon described above for the biomarkers suggests clusters and subclusters with consistent patterns of joint evolution of CD4 and VL; interpreting these patterns can shed light on the biology driving HIV disease progression. The ensemble results provide further evidence of the association between lower CD4 levels and higher VL levels. In addition, the higher VL groups (univariate VL cluster groups 1 and 3 from Fig. 3) are associated strongly with the consensus group 1 (predominated by low CD4 levels), save for the few profiles that are distinct within the high CD4 subcluster (consensus group 4). These analyses strengthen the evidence of the association between patterns of the time courses of CD4 counts and of VL and thereby may provide insight regarding the dynamic nature of these markers of HIV progression.

## Summary

The behavior of biomarkers over time is the primary information available for monitoring disease progression and response to intervention in a patient population, and requires profile clustering—finding groups with similar prognoses in unlabeled data. Above, we demonstrated that shape-respecting distance measures are useful in meeting this goal. We categorize prognoses of HIV infection into different groups using biomarker trajectory data from a cohort of people at different stages of HIV infection, using our proposed method combining machine learning using shape-respecting distances with ensemble clustering. We showed that it is possible to distinguish between groups of study subjects based on their bivariate longitudinal profiles and to make inference on their likely disease stage, without knowledge of the infection time. Analyses of such data were complicated because of their internal (rather than chronologic) time referencing, sparsity, irregular time measurements, and variable follow up as well as detection limits for VL. We analyzed distance measures that retain measurement ordering and provide information on global shape of the profiles, but allow flexibility of time axis to accommodate the fact that times of infection for cohort members were unknown. We combined information from both markers using ensemble learning methods, which accommodate the non-metric distances used in profile clustering by operating directly on the cluster dendrograms via ultrametric measures that combine across individual biomarker cluster results for information synthesis. Our results support the notion that within the cohort of people in Botswana with prevalent HIV infection, was a small set of recently infected individuals. These results demonstrate the usefulness of machine learning tools applied to longitudinal profile data to obtain insights about progression of HIV infection. The methods provide a unique modeling tool for leveraging multiple marker profiles, and could prove an important tool in analyzing longitudinal data in many infectious disease settings where times of infection are often unknown.

## Supplementary Information

Below is the link to the electronic supplementary material.

Supplementary file 1 (pdf 8 KB)

Supplementary file 2 (pdf 16 KB)

## Data Availability

All scripting used for simulated data generation is available in the Github repository at MLLynch10/HIVManuscriptCodeSimulation.
