# Supplementary figures and images for: Ensemble clustering of longitudinal bivariate HIV biomarker profiles to group patients by patterns of disease progression
Source: Int J Data Sci Anal. 2022 May 4;14(3):305–18. doi: 10.1007/s41060-022-00323-2 (PMC9064718; doi:10.1007/s41060-022-00323-2)

**Cylinder**

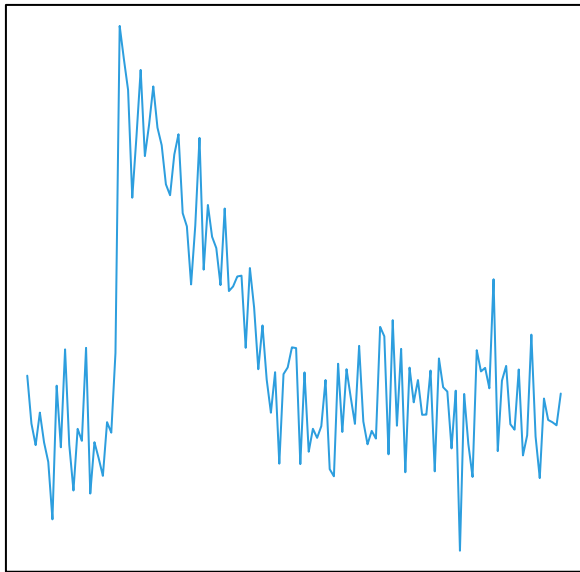

**Bell**

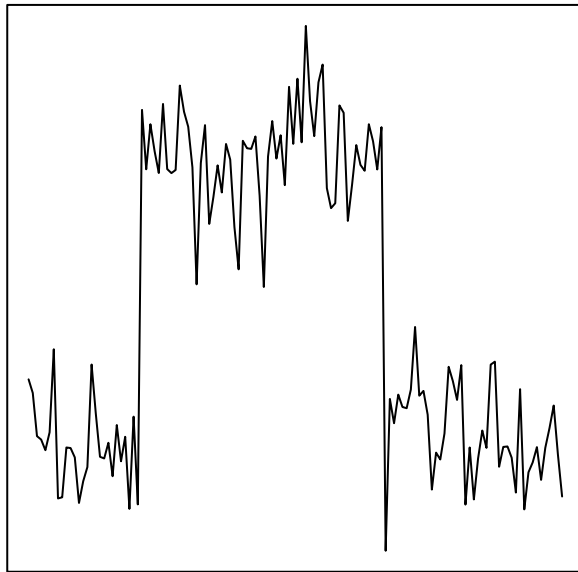

**Funnel**

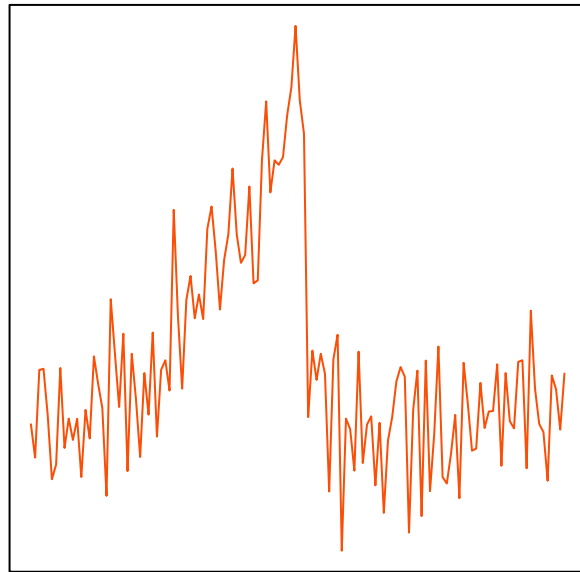

Supplement: Supplementary file 1 — (pdf 8 KB) [file 41060_2022_323_MOESM1_ESM.pdf]

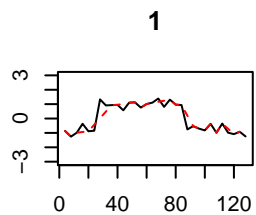

1

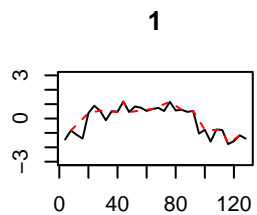

2

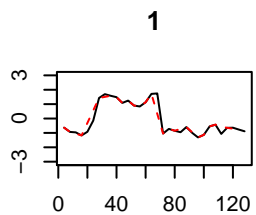

3

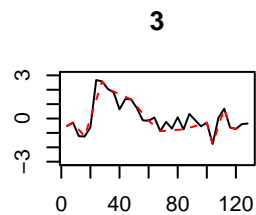

4

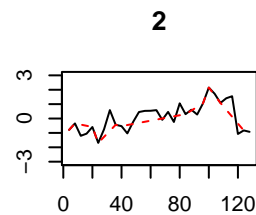

5

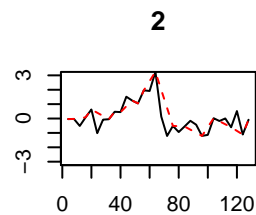

6

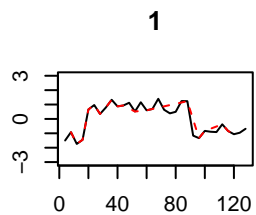

7

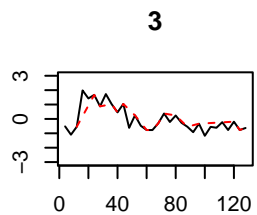

8

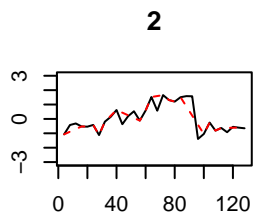

9

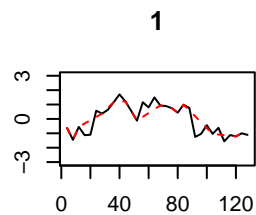

10

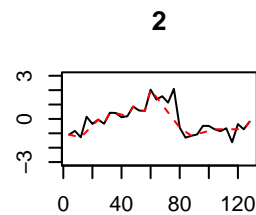

11

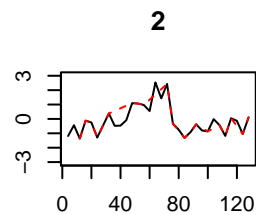

12

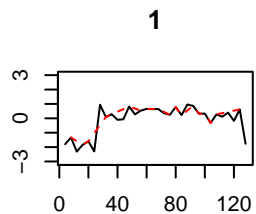

13

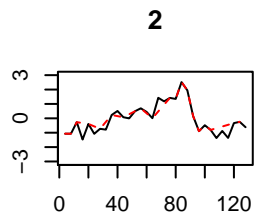

14

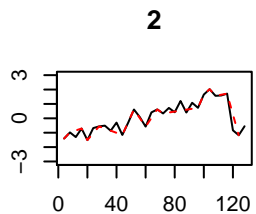

15

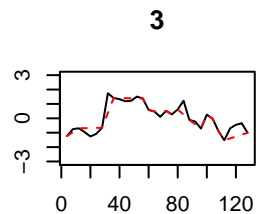

16

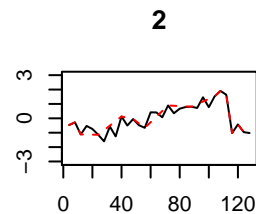

17

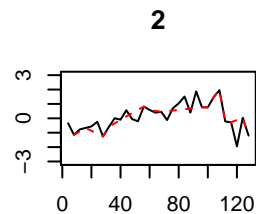

18

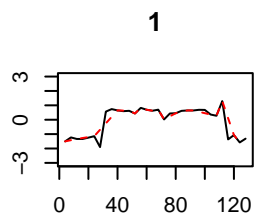

19

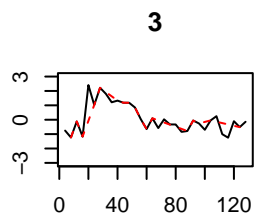

20

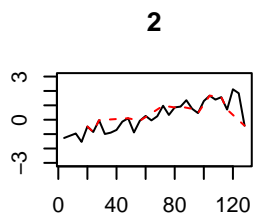

21

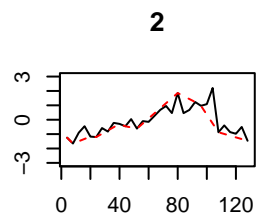

22

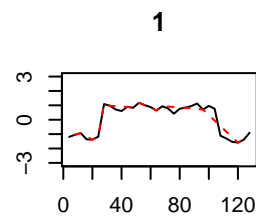

23

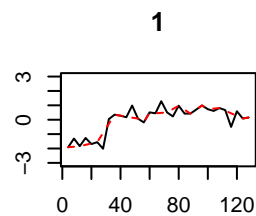

24

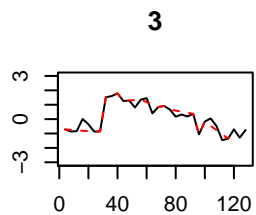

25

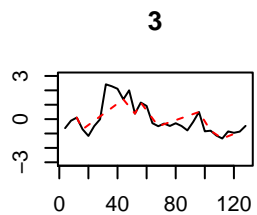

26

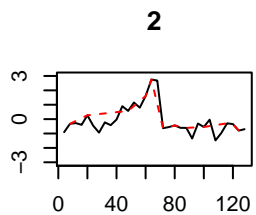

27

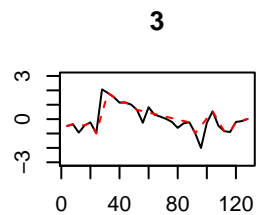

28

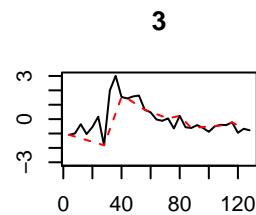

29

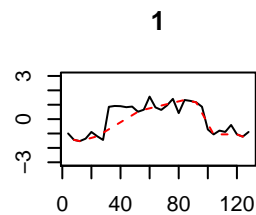

30

Supplement: Supplementary file 2 — (pdf 16 KB) [file 41060_2022_323_MOESM2_ESM.pdf]
